# Transcriptome Sequencing Analysis of Genes Associated with Different Developmental Periods of the Ovarian Follicle in the Duolang Sheep

**DOI:** 10.3390/genes15111394

**Published:** 2024-10-29

**Authors:** Chengqian Wang, Hang Yan, Wen Hao, Fugui Li, Tianci Liu, Hui’e Wang

**Affiliations:** 1 College of Animal Science and Technology, Tarim University, Alar 843300, China; wcqdky@126.com (C.W.); 18326416893@163.com (H.Y.);; 2 Key Laboratory of Livestock and Forage Resources Utilization Around Tarim, Ministry of Agriculture and Rural Affairs, Alar 843300, China

**Keywords:** Duolang sheep, transcriptome sequencing, ovarian follicles, RT-qPCR

## Abstract

Background: The ovaries are crucial reproductive organs in female mammals, directly influencing the reproductive efficiency and productivity of these animals. The Duolang sheep, native to Xinjiang, is known for its rapid growth and high fertility. However, the mechanisms underlying ovarian follicle development and regulation in sheep remain unclear. Methods: Employing transcriptome sequencing technology, this study methodically analyzed ovaries from sheep across various estrous cycles to uncover key genes and signaling pathways that play a role in the development of ovarian follicles. Results: The results indicated that a total of 130, 183, and 175 differentially expressed genes were identified in the DTA/DTB, DTB/DTC, and DTA/DTC groups, respectively. Key genes like *BAG3*, *GDF5*, *RHOB*, *RUNX2*, *LGALS3*, and *CDH1*, along with pathways such as endoplasmic reticulum protein processing, the NOTCH signaling pathway, and the MAPK signaling pathway, were found to be involved. RT-qPCR confirmed the differential expression of *BAG3*, *RHOB*, and *RUNX2*. Conclusions: This research provides insights into the molecular mechanisms of ovarian follicle development and a basis for enhancing the reproductive performance of Duolang sheep.

## 1. Introduction

As society evolves and living standards improve, the demand for meat consumption continues to rise. Lamb, as one of the primary sources of meat, has become a popular choice among consumers. The Chinese market has experienced an increase in demand for mutton due to its delicious flavor, low steroid content [1], and high nutritional value. However, there is a significant disparity between the import and export volumes of mutton in China. To address this issue, enhancing the reproductive performance of sheep and increasing production numbers are effective solutions.

The 2021 ‘National Catalogue of Livestock and Poultry Genetic Resources’ reported 89 sheep breeds in China. The Duolang sheep breed is primarily raised in Maigaiti County and the surrounding regions of Kashgar City, Xinjiang. This breed offers several advantages, including its large size, high meat yield, delicious meat, early sexual maturity, and strong reproductive capabilities. Duolang rams achieve sexual maturity at the age of 6 to 7 months, while ewes reach this milestone around 6 months old. The age at which they are typically introduced to trial mating is approximately 9 months. The estrus time is about 15 days to 18 days, and the pregnancy time of ewes is about 150 days. Two lambs are born each year, with an average survival rate of about 150% and a twinning rate of 50%. The average weight of adult rams is 80 kg and spans up to 140 kg. The average weight of adult ewes is 60 kg, spanning up to 110 kg, and the average slaughter rate is 53~60%. This breed is particularly suitable for investigating the reproductive capabilities of sheep, given its excellent potential for meat and fat production [2].

The condition of the ovaries is key to a female mammal’s reproductive success, as it is vital for oocyte growth and follicular ovulation through hormone regulation [3]. Follicles, found in the ovary’s cortex, are made up of granulosa cells surrounding oocytes and go through recruitment, selection, and maturation phases, with five distinct developmental stages [4]. These follicles are influenced by transcription factors and signaling pathways [5,6] and most undergo apoptosis, with only a few maturing and ovulating, affecting reproductive efficiency [7]. Thus, understanding the molecular mechanisms of follicle development and apoptosis in the ovaries is crucial for insights into regulating reproductive performance in sheep.

In recent years, advancements in transcriptome sequencing technology have significantly enhanced our understanding of reproductive performance and ovarian follicular development in sheep. Tang’s study utilized transcriptome sequencing to identify several key genes, such as *STAR*, *HYAL2*, *COX7A1*, *QCR10*, *GDF9*, *CD81*, and *AIFM1*, in the ovaries of single-lambing and multi-lambing ewes, suggesting a possible link to the multi-lambing trait [8]. Furthermore, three crucial signaling pathways involved in the development of the multi-lambing trait were identified: ovarian steroid hormone synthesis, oxidative phosphorylation, and ribosome biogenesis. Wang et al. [9] identified 5409 differentially expressed genes in the ovaries of sheep subjected to superovulation and estrus synchronization. Among these, 2164 genes were up-regulated, while 3245 genes were down-regulated. They predicted that genes such as *TNFAIP6*, *CYP11A1*, *COL3A1*, *RPS8*, *ACTA2*, and *RPL7* may play significant roles in regulating follicular development. Additionally, they determined that signaling pathways related to amino acid biosynthesis, ovarian steroid production, and carbon metabolism are directly associated with follicular development and maturation. Currently, research on transcriptomics related to sheep reproduction has primarily focused on identifying genes specific to ovarian development across two or more different breeds. However, there is a lack of studies examining the differences in the ovarian follicle dynamic transcriptome profiles at various developmental stages in Duolang sheep.

Inserting a progesterone-containing intravaginal device into a ewe’s vagina for a period of 9 to 14 days effectively synchronizes the estrous cycle, mimicking the luteal phase of the reproductive cycle as if the animal were not pregnant. Once the plug is removed, this triggers the follicular phase due to the withdrawal of progesterone [10]. In this study, Duolang sheep were treated with this method to synchronize estrus, and their ovaries were examined on the 11th, 13th, and 15th days to record the pre-estrus, estrus, and post-estrus phases, respectively. Transcriptome sequencing was used to explore the expression and regulation of protein-coding RNA at these critical developmental stages of the ovaries. The selection of these specific days was strategic, as it allowed for a comprehensive analysis of the molecular mechanisms across the entire estrous cycle, from the pre-estrus period (11th day), through the peak of estrus (13th day), to the post-estrus phase (15th day). This study aimed to discover the key genes behind ovarian follicle development in Duolang sheep in order to understand its molecular mechanisms and improve this breed’s reproductive capabilities.

## 2. Materials and Methods

### 2.1. Collection of Tissue Samples and Ethics Statement

**Figure 1:**
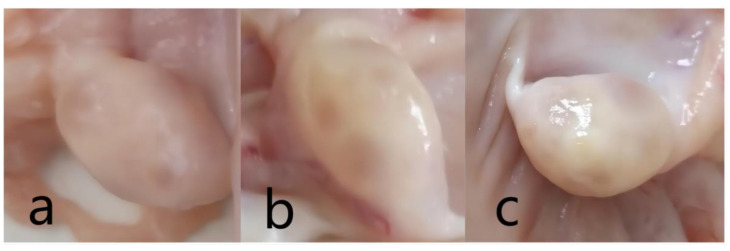
Ovary at different developmental stages. (**a**) Ovarian tissue on day 11; (**b**) ovarian tissue on day 13; (**c**) ovarian tissue on day 15.

Our experiment was conducted in accordance with the guidelines for ethical review of laboratory animal welfare in the People’s Republic of China National Standard GB/T 35892-2018 and received approval from the Ethics Committee of Science and Technology of Tarim University (approval no. 2024065). Nine healthy adult multiparous Duolang sheep, aged 2–3 years old and weighing 50 ± 5 kg, were selected for the experiment. They were quarantined in the experimental station of Tarim University one month before the experiment. They received unified feeding management, health observations, and necessary deworming treatment. After the quarantine, all the sheep were confirmed to be in good health and not pregnant, in non-estrus, which met the experimental standards. Then, they were fed alfalfa hay, carrots, concentrated corn and soybean meal, salt, and bone meal diets every day and drank clean and sufficient water. The sheep house was kept dry and clean, the manure was cleaned in a timely manner, and the appropriate ventilation was properly established to ensure a suitable living environment. All the sheep were simultaneously administered a CIDR Silica Progesterone Suppository (0.3 mg) (CIDR). The day of insertion was marked as day 0, and the treatment lasted for 13 days. At the end of day 13, the CIDR was removed, and the sheep were given an intramuscular injection of 0.2 mg of PG. On the morning of the 11th day, three sheep were slaughtered and designated as DTA1, DTA2, and DTA3. On the morning of the 13th day, another three sheep were slaughtered and labeled as DTB1, DTB2, and DTB3. The final three sheep were slaughtered on the morning of the 15th day and marked as DTC1, DTC2, and DTC3, respectively. The test sheep were euthanized on the designated days, and their ovaries were promptly removed within five minutes. The diameter and number of follicles on the surface of both ovaries were measured and recorded after the surrounding fat and connective tissue were excised. The surface of the ovaries was washed with saline at 37 °C until the wash water was colorless to eliminate any blood. Subsequently, the ovaries were rapidly frozen in liquid nitrogen and stored in a freezer at −80 °C until RNA extraction. Figure 1 illustrates the ovaries at different developmental stages.

### 2.2. RNA Extraction, Library Construction, and Illumina Sequencing

RNA library construction and deep sequencing were conducted by Beijing Novo Biotechnology Company (Beijing, China). The total RNA was extracted using the Tiangen RNA extraction kit (DP451, Tiangen Biochemical Technology Co., Ltd., Beijing, China). The samples collected on the 11th, 13th, and 15th days were designated as belonging to the DTA group (DTA1, 2, and 3), the DTB group (DTB1, 2, and 3), and the DTC group (DTC1, 2, and 3). RNA integrity was assessed using 1% agarose gel electrophoresis and an Agilent 2100 Bioanalyzer (Agilent Technologies, Santa Clara, CA, USA). RNA purity and concentration were quantified precisely using a NanoPhotometer spectrophotometer (IMPLEN, Westlakevillage, CA, USA) and a Qubit^®^ 2.0 fluorometer (Life Technologies, Carlsbad, CA, USA) to ensure accurate measurement of the RNA quality and concentration. The cDNA library was prepared using the NEB Next^®^ Ultra^TM^ RNA Library Preparation Kit (New England Biolab, Ipswick, MA, USA) in accordance with the instructions provided. The preparation process was as follows: First, ribosomal RNA was removed from the total RNA to isolate the mRNA. The resulting mRNA was then randomly fragmented using divalent cations in NEB Fragmentation Buffer. The first strand of cDNA was synthesized in the M-MuLV reverse transcriptase system, utilizing the fragmented mRNA as a template and random oligonucleotides as the primers. This was followed by the degradation of the RNA strand with RNase H and the synthesis of the second strand of cDNA using dNTPs under a DNA polymerase I system. The purified double-stranded cDNAs were end-repaired, A-tailed, and ligated to sequencing adapters. cDNAs of approximately 200 bp were screened using AMPure XP beads and PCR-amplified, and the PCR products were purified again with AMPure XP beads to obtain the final library. Following library preparation, the quality of the library was assessed on the Agilent Bioanalyzer 2100 system after the addition of aptamers. The sequencing of the nine libraries was performed using the Illumina HiSeq 2500 platform, which is manufactured by Illumina, Inc., headquartered in San Diego, CA, USA. The sequencing was carried out with a read length of 125 base pairs for the paired-end 150 base pair setup.

In order to ensure the quality and reliability of the data analysis, we removed low-quality reads to obtain clean data. Hisat2 (v2.0.5) was used to align the filtered high-quality clean data with a reference sheep genome (http://asia.ensembl.org/Ovis_aries_rambouillet/Info/Index (accessed on 21 October 2024)).

### 2.3. Differential Expression Analysis

We utilized Feature Counts (v1.5.0-p3) software to determine the number of reads mapped to each gene using Hisat2. We then combined these data with the gene length information to calculate the number of fragments per kilobase (FPKM) for each gene, allowing us to quantify the amount of non-gene expression. Cuffdiff (v2.1.1) was employed to compute the FPKM of the genes in each sample. Subsequently, differential expression analysis of the gene expression data was performed using a negative binomial distribution model in the DESeq2 R package (v1.16.1) [11]. The Benjamini–Hochberg procedure was applied to adjust the *p*-values and control for false discovery rates, identifying genes with *p*-values < 0.05, which were designated as differentially expressed genes.

### 2.4. Functional Annotation and PPI Analysis

Functional annotation and pathway enrichment analysis of the differentially expressed genes (DEGs) were conducted using the Gene Ontology (GO) database [12] and the Kyoto Encyclopedia of Genes and Genomes (KEGG) database [13] to elucidate the primary biological functions of the DEGs and the key signal transduction pathways involved. Simultaneously, we performed protein–protein interaction (PPI) analysis of the DEGs based on the STRING database to gain a deeper understanding the relationships between the proteins [14].

### 2.5. Verification of the Reliability of the RNA-Seq Data

To verify the accuracy of the key differential genes identified through the transcriptome sequencing data, we analyzed the relative expression levels of key genes using RT-qPCR. GAPDH served as the internal reference gene, and the primers for fluorescence quantification were designed by using the software Primer Premier 5.0 software. The primers designed were specifically compared against the NCBI database (http://www.ncbi.nlm.nih.gov/). Once the comparison was validated, the primers were sent to Beijing Qingke Biotechnology Co., Ltd. (Beijing, China) for synthesis.

## 3. Results

### 3.1. Morphological Analysis of Ovarian Follicles in Sheep

On day 11 of the study, ovarian follicle development in the sheep was subtle, with an average of 5.33 dominant follicles. By day 13, in the midst of estrus, the follicle numbers started to rise, peaking at an average of 7.33 dominant follicles. However, by day 15, as the sheep entered late estrus, the follicle numbers declined due to atresia, and the average number of dominant follicles dropped to 4, still at a peak level.

### 3.2. Results and Analysis of Sequencing Data

**Figure 2:**
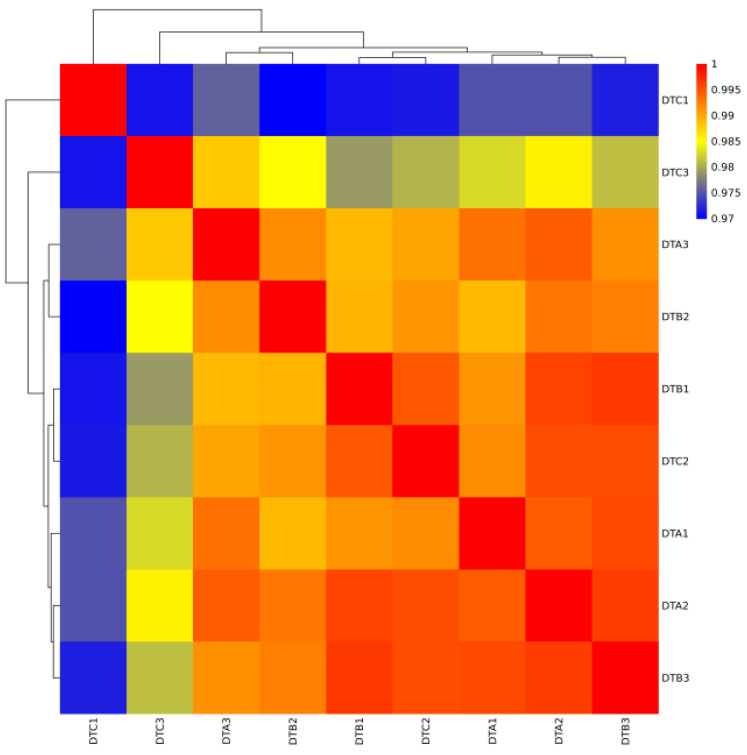
Heat map of ovarian correlation coefficients at different developmental stages in Duolang sheep.

**Figure 3:**
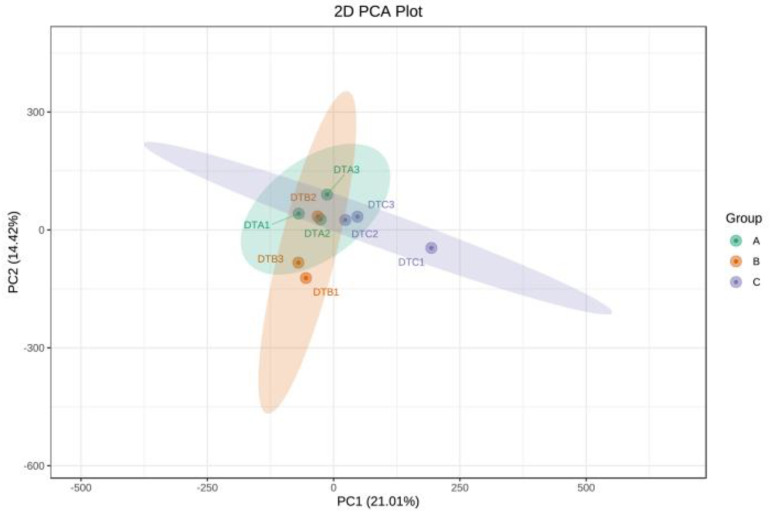
PCA.

The analysis of the RNA-seq results revealed that a total of 375,569,273 raw reads were obtained, of which 368,931,170 were classified as high-quality reads. Following quality control, it was determined that the percentage of high-quality data exceeded 94.15% (Table 1). Comparative analysis with the reference gene set indicated that the comparison efficiency ranged from 79.36% to 84.22%, while the unique coverage for each sample varied from 74.78% to 79.39% (Table 2). These findings suggest that the data utilization was satisfactory and that the selected reference gene met the requirements for subsequent data analysis. The results of Pearson’s correlation coefficient (r) analysis indicated that the minimum r value was 0.97 for each sample (Figure 2). This finding suggests that the sample selection was appropriate, the experimental reproducibility was high, and the results were reliable. Additionally, principal component analysis (PCA) offered insights into the relationships among the various developmental stages of the ovaries in Duolang sheep. The analysis revealed that the first principal component (PC1) accounted for 21.01% of the variance, while the second principal component (PC2) accounted for 14.42% (Figure 3), demonstrating strong biological reproducibility among the experimental samples.

### 3.3. Differential Expression Analysis Results

**Figure 4:**
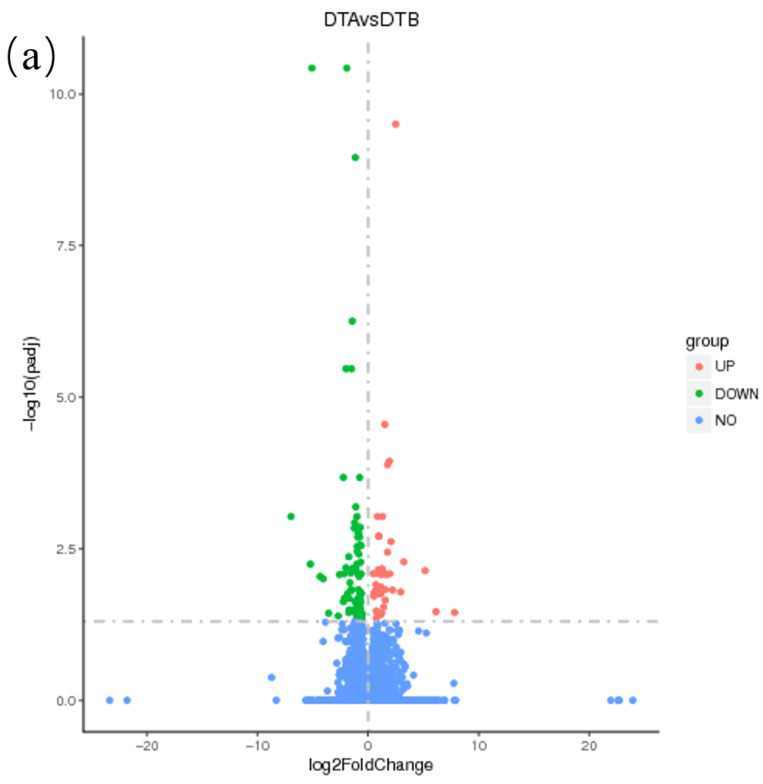

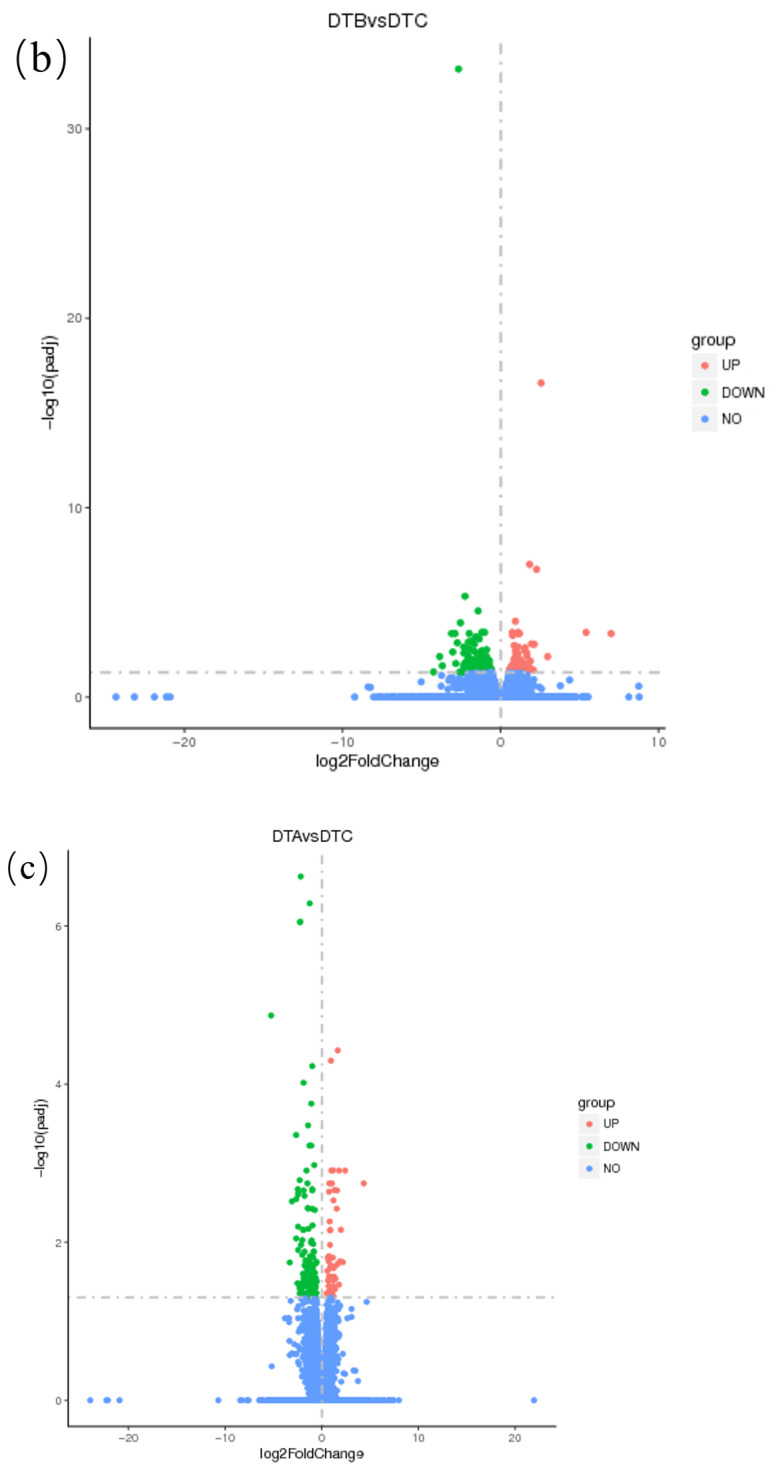
Volcanic map of the number of genes differing between groups of ovaries at different developmental stages in Duolang sheep. UP: Up-regulated, that is, the gene expression of the experimental group or the treatment group was significantly higher than that of the control group. DOWN: Indicates down-regulation, that is, the gene expression of the experimental group or the treatment group was significantly lower than that of the control group. NO: There was no significant difference (not significant); that is, there was no significant difference in the gene expression between the experimental group and the control group. The dotted line represents the screening threshold, -lg (0.05) = 1.3. (**a**) DTA/DTB. (**b**) DTB/DTC. (**c**) DTA/DTC.

**Figure 5:**
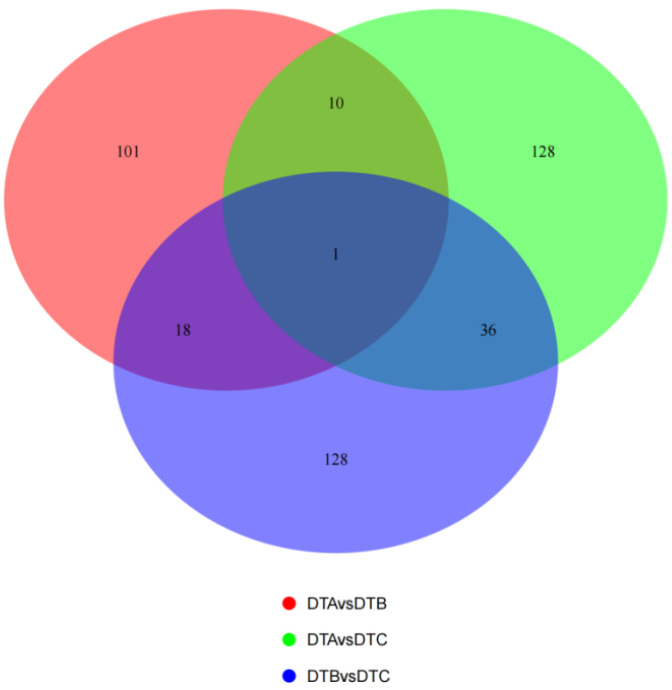
Venn diagram of genes differing between the three groups.

The FPKM algorithm was used to standardize the difference in the gene transcription levels. The threshold was established as log2|Fold Change|> 1, with a significance level of *p* < 0.05, for screening. The results indicated that there were 130 differentially expressed genes, comprising 41 up-regulated genes and 89 down-regulated genes in the DTA/DTB group. In the DTB/DTC group, 183 differentially expressed genes were identified, including 80 up-regulated genes and 103 down-regulated genes. In the DTA/DTC group, there were 175 differentially expressed genes, consisting of 57 up-regulated genes and 118 down-regulated genes (Figure 4). A total of 30,186 expressed genes were detected in the ovarian tissues, with 101, 128, and 128 uniquely expressed genes in each respective group, and only 1 gene was expressed across all three groups (Figure 5).

**Figure 6:**
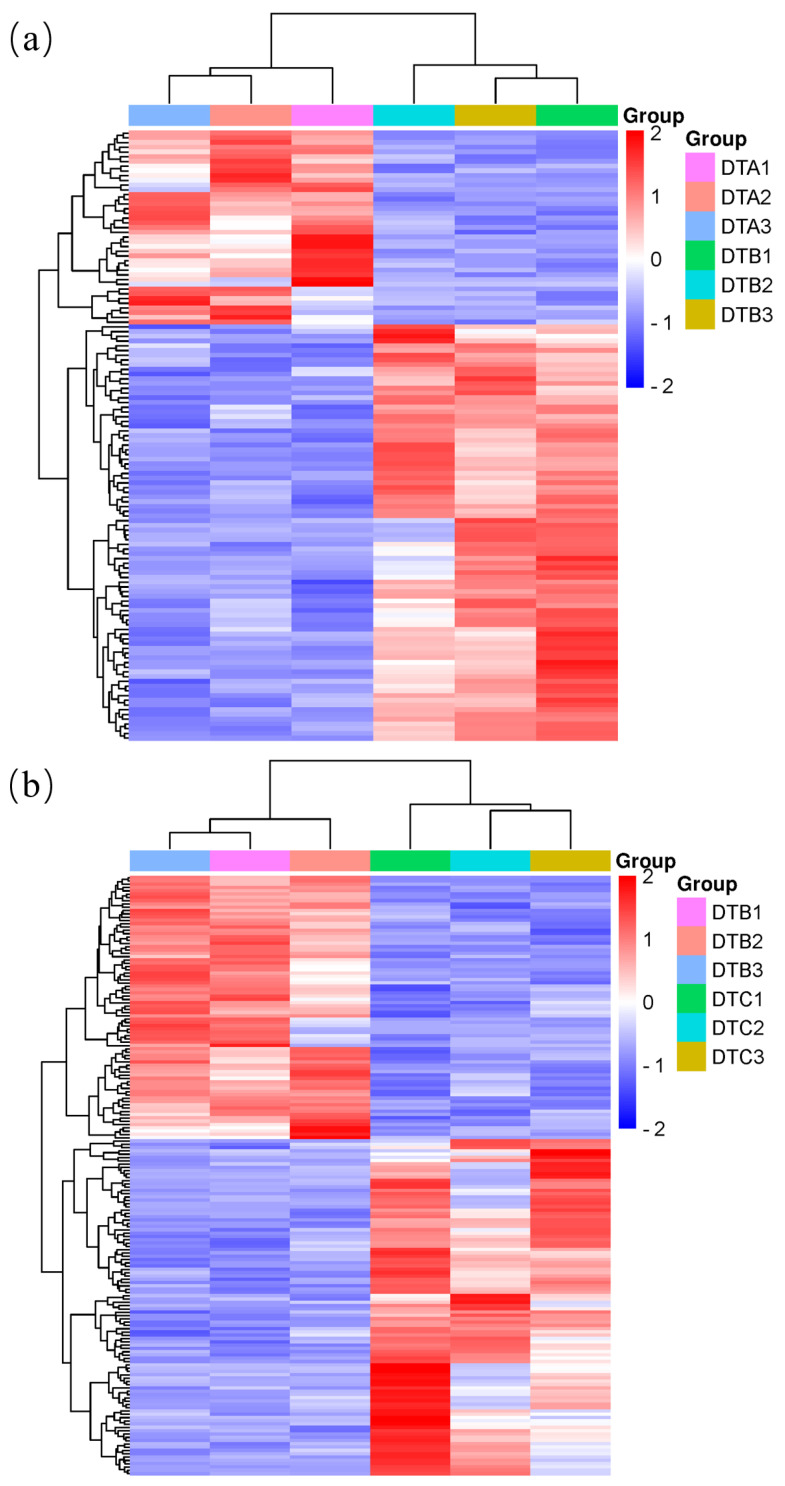

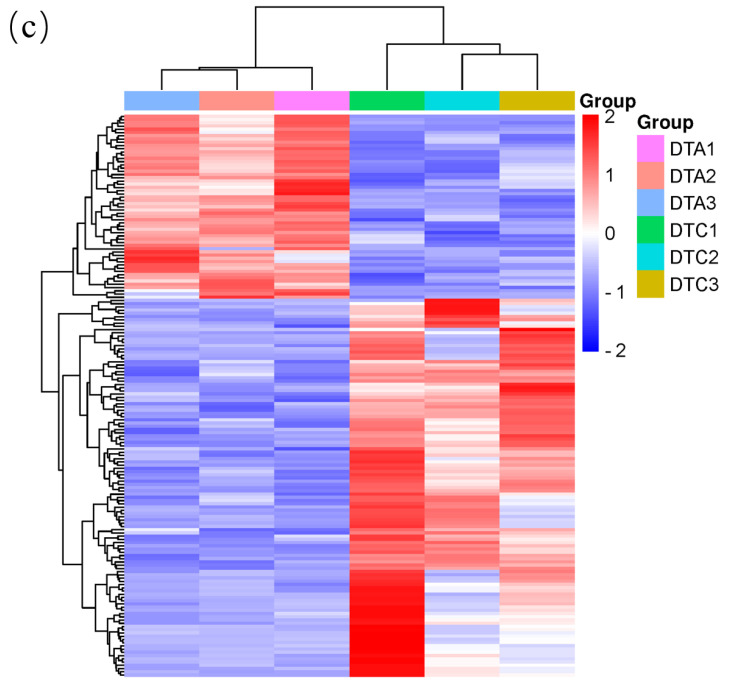
Clustering analysis of differentially expressed genes among groups of ovaries at different developmental stages in Duolang sheep. (**a**) DTA/DTB; (**b**) DTB/DTC; (**c**) DTA/DTC.

Hierarchical clustering analysis was conducted on all the differentially expressed genes screened based on their expression levels and the relationships between genes. This analysis aimed to explore the correlation of the gene expression patterns across different samples and is presented in the form of heat maps, where each column represents a sample and each row represents a gene (Figure 6).

### 3.4. Functional Annotation and Enrichment Analysis of Differential Genes

**Figure 7:**
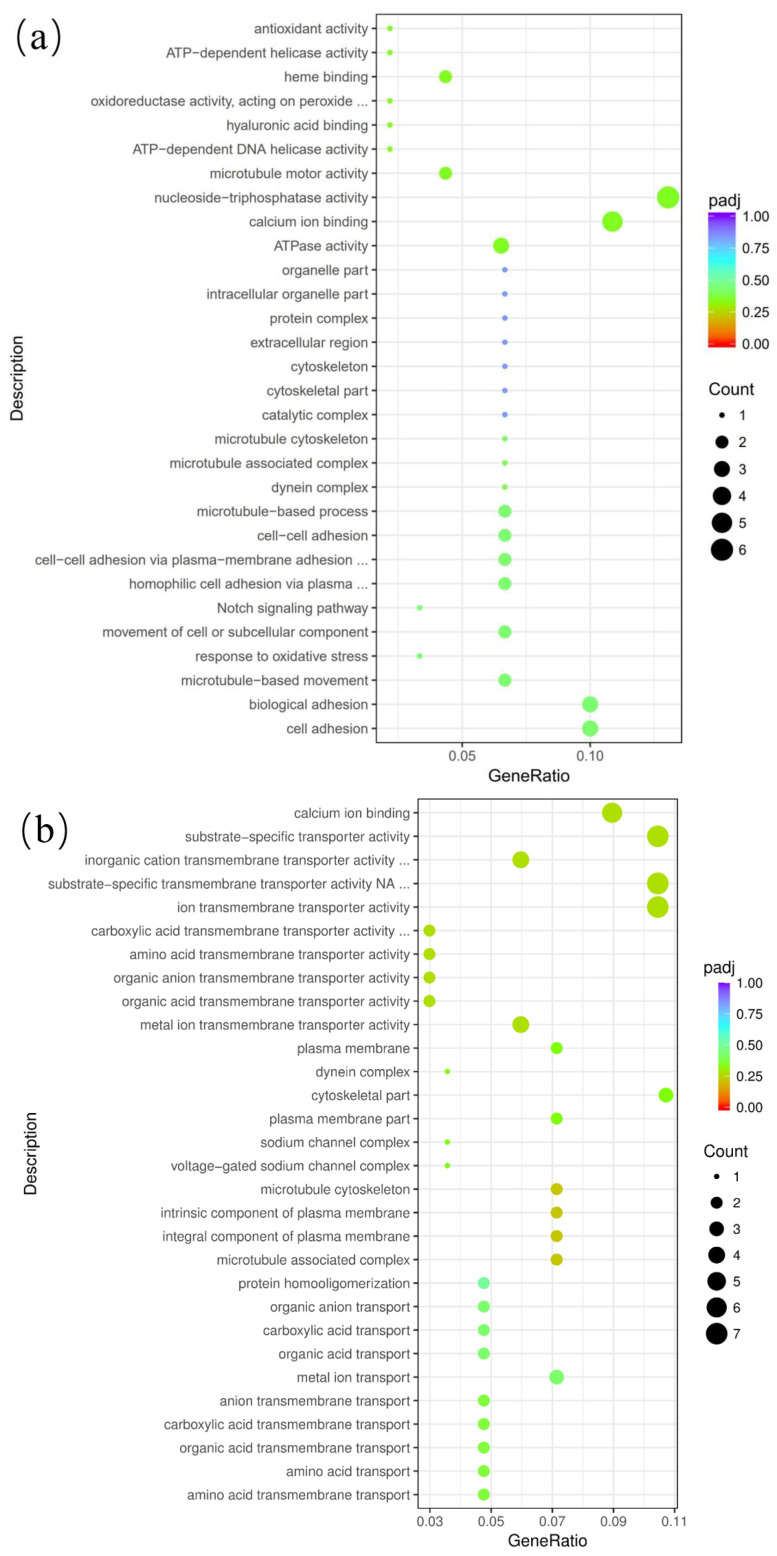

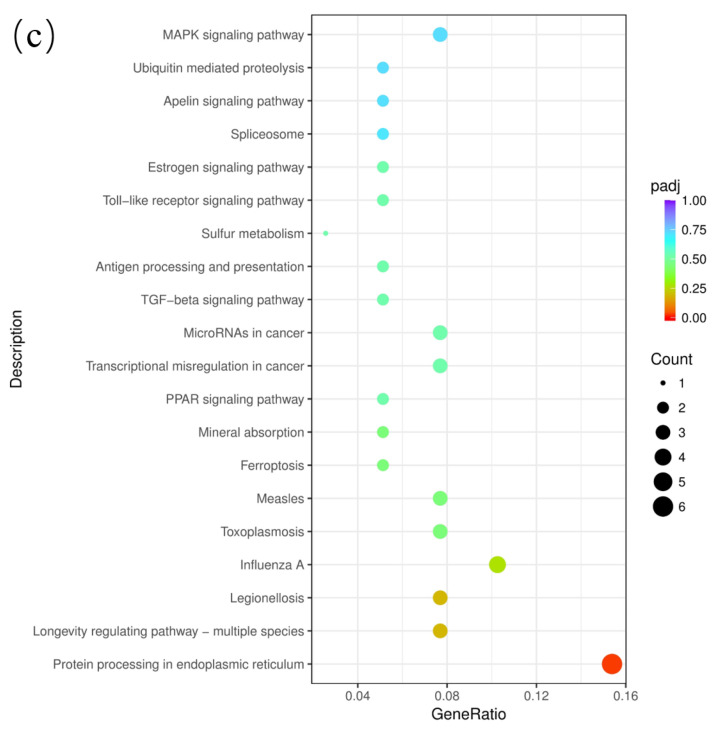
Functional analysis of differential genes in GO between the three groups. Padj: Represents the corrected *p*-value (*p*-adjusted), which is used to measure the statistical significance of the enrichment analysis results. The bubble color is usually used to represent the size of the padj value. The darker the color (i.e., red), the lower the padj value, and the more significant the enrichment. Count: Represents the number of differential genes enriched in a specific pathway or functional category. The size of the bubble area is usually used to represent the number of counts; that is, the larger the bubble, the greater the number of differential genes enriched in said pathway or functional category. (**a**) DTA/DTB; (**b**) DTB/DTC; (**c**) DTA/DTC.

Enrichment and analysis of differentially expressed genes at various developmental stages of the ovaries in Duolang sheep through GO functional annotation are presented. Figure 7 illustrates the 30 GO terms with the highest enrichment in each group. In the DTA/DTB group, four entries were significantly enriched (*p* < 0.05), including cell adhesion and bioadhesion (Figure 7a). The DTB/DTC exhibited 11 significantly enriched entries (*p* < 0.05), such as amino acid transmembrane transport and ion transmembrane transport activity (Figure 7b). Additionally, the DTA/DTC group revealed six significantly enriched entries (*p* < 0.05), including cell growth and regulation of cellular component organization (Figure 7c).

**Figure 8:**
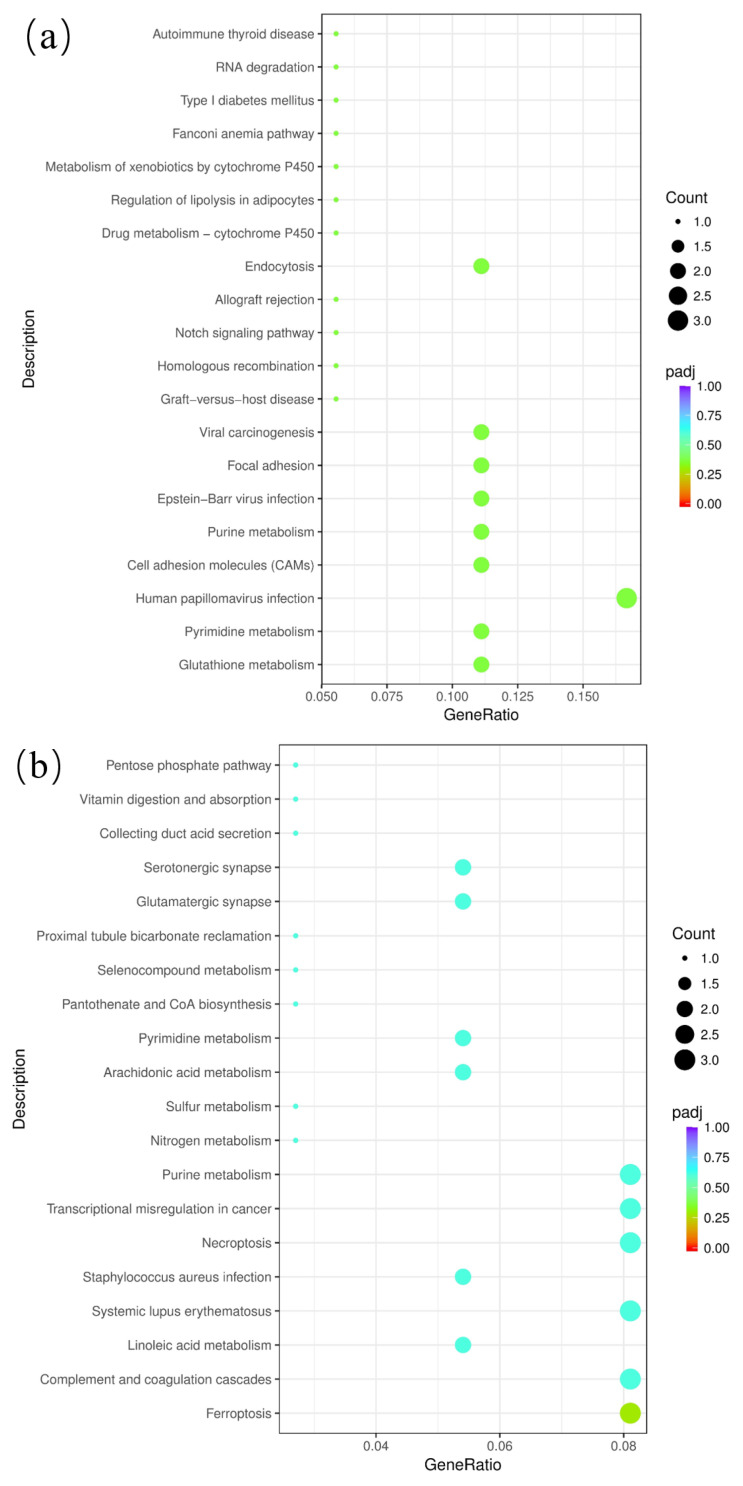

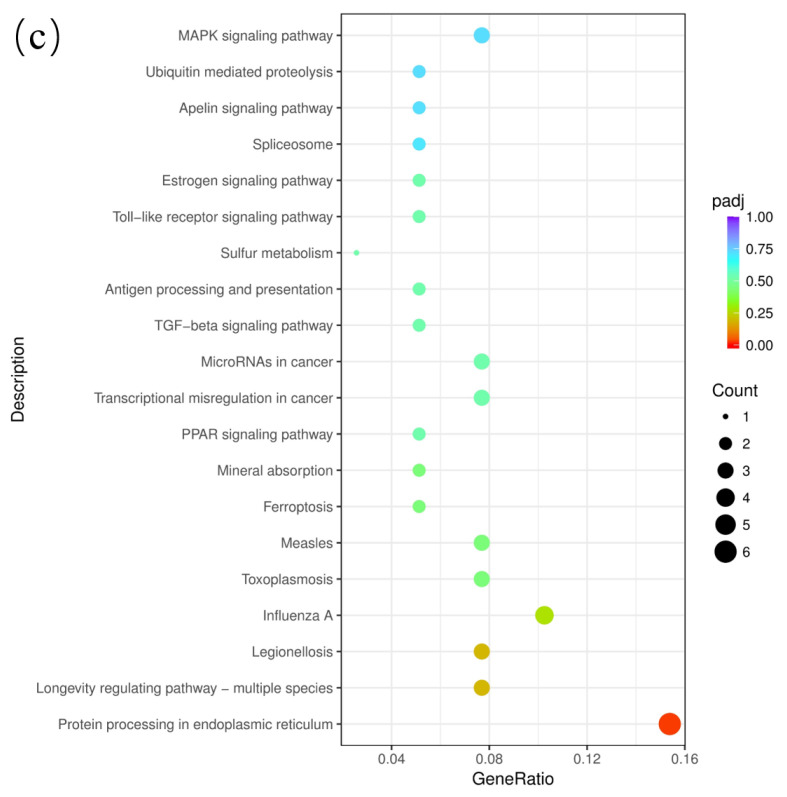
KEGG enrichment analysis of differential genes between the three groups. Padj: Represents the corrected *p*-value (*p*-adjusted), which is used to measure the statistical significance of the enrichment analysis results. The bubble color is usually used to represent the size of the padj value. The darker the color (i.e., red), the lower the padj value, and the more significant the enrichment. Count: Represents the number of differential genes enriched in a specific pathway or functional category. The size of the bubble area is usually used to represent the number of counts; that is, the larger the bubble, the greater the number of differential genes enriched in said pathway or functional category. (**a**) DTA/DTB; (**b**) DTB/DTC; (**c**) DTA/DTC.

Through KEGG analysis, we gained insights into the significantly enriched pathways of the differentially expressed genes at various developmental stages. Figure 8 illustrates the 20 signaling pathways with the highest enrichment of these genes in each group. In the DTA/DTB group, three significantly enriched signaling pathways were identified: glutathione metabolism, pyrimidine metabolism, and human papillomavirus infection. There were five significantly enriched signaling pathways identified in the DTB/DTC group, including ferroptosis and the linoleic acid metabolism signaling pathway, among others. In the DTA/DTC group, eight significantly enriched signaling pathways were identified, including processing in the endoplasmic reticulum and the mineral absorption signaling pathway, among others (Figure 8).

**Figure 9:**
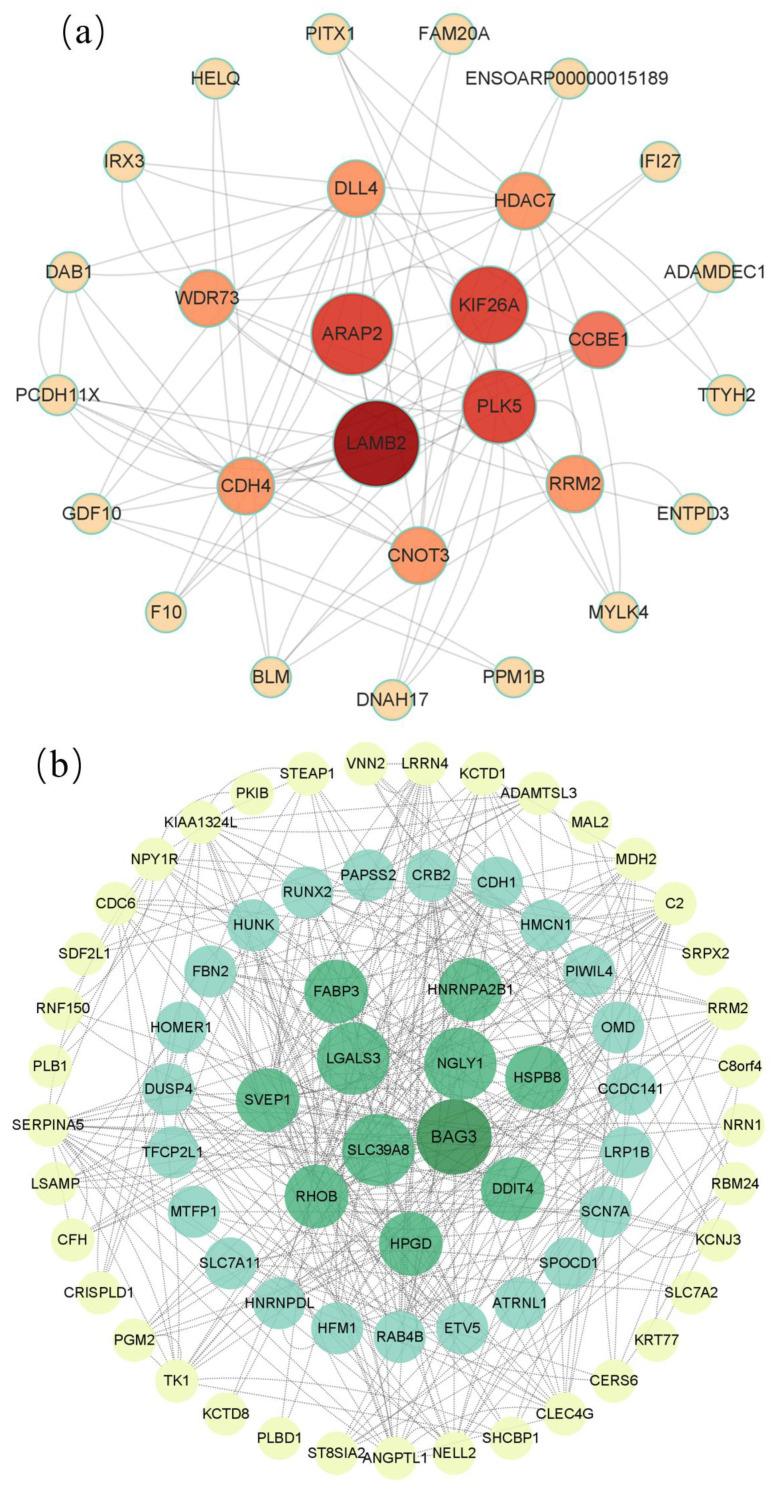

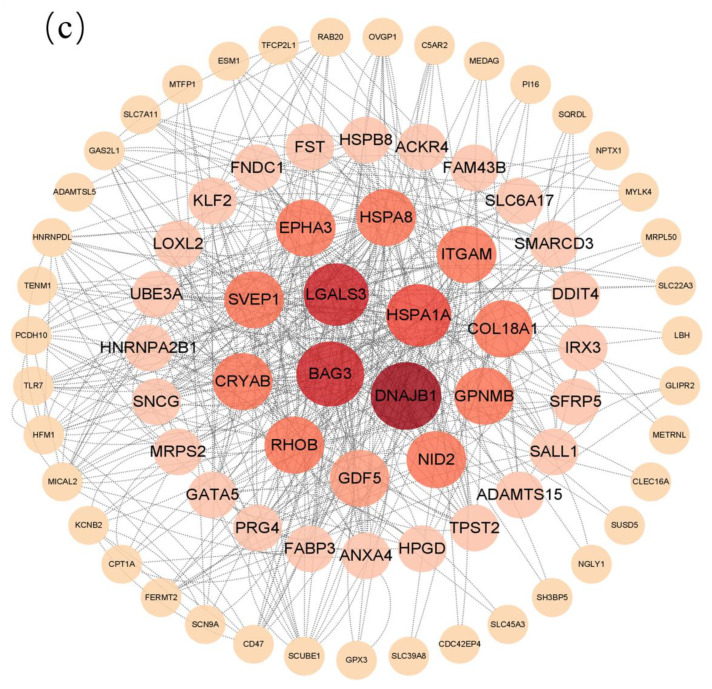
Protein interactions of differential gene PPI networks among the three groups. The depth of the color is mapped according to the score value of the interaction. The darker the color, the stronger the evidence of the interaction or the higher the credibility of the interaction. (**a**) DTA/DTB; (**b**) DTB/DTC; (**c**) DTA/DTC.

Our PPI analysis aimed to reveal the interrelationship between differentially expressed genes at various developmental stages of the ovaries. Figure 5 displays the top-ranked core genes filtered based on betweenness (Figure 9).

Finally, a combination of functional annotation, pathway enrichment, PPI analysis, and a literature review was used to initially screen for genes associated with ovarian follicle development: *BAG3*, *RHOB*, *GDF5*, *RUNX2*, *LGALS3*, and *CDH1* (Table 3).

### 3.5. Identification of Key Differential Candidate Genes

**Figure 10:**
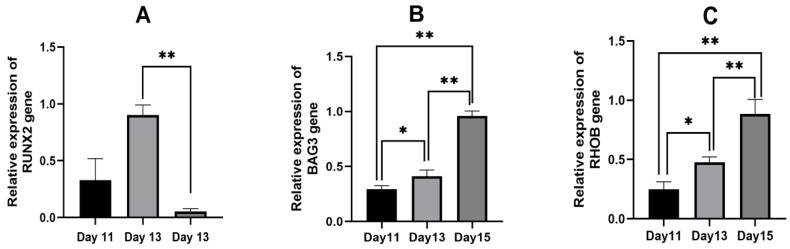
Experimental verification of gene expression levels across three time points using RT-qPCR (*n* = 3). Note: The *Y*-axis on the left side of the histogram represents the gene expression level according to RT-qPCR, and the right *Y*-axis represents the standard value of FPKM based on transcription. * means significant difference (*p* < 0.05); ** indicated that the difference was extremely significant (*p* < 0.01).

Having thoroughly assessed the biological relevance of six genes during various estrus stages and the significance of their transcriptional alterations, due to constraints in experimental resources and time, we chose *BAG3*, *RHOB*, and *RUNX2*—genes implicated in ovarian development—for detailed qPCR analysis. The list of primers is provided in Table 4. The expression of the *RUNX2* gene was significantly higher on day 13 compared to day 15 (*p* < 0.01). Additionally, the expression of the *BAG3* gene was significantly elevated on day 15 relative to days 11 and 13 (*p* < 0.01), and the expression of the *RHOB* gene was also significantly higher on day 15 than on days 11 and 13 (*p* < 0.01) (Figure 10).

## 4. Discussion

The developmental status of ovarian follicles is a crucial factor in determining the reproductive performance of female mammals [15]. In most high-yielding sheep, the developmental status of the ovaries and the number of mature follicles are significantly superior to those in low-yielding sheep. Therefore, analyzing the mechanisms underlying the growth and development of ovarian follicles is an effective approach to enhancing the reproductive performance of sheep. In this study, we compared and analyzed the gene expression differences in the ovaries of Duolang sheep at various developmental stages using transcriptome sequencing technology. A total of 422 differentially expressed genes were identified following rigorous screening based on established thresholds. Subsequent functional annotation and KEGG enrichment analysis revealed that these differential genes were primarily enriched in transmembrane transport, cell adhesion, cell growth, and other related functional categories, as well as in pathways associated with endoplasmic reticulum protein processing, cell adhesion molecules, and NOTCH and MAPK signaling. By integrating these analyses with a literature review, we further identified that the genes *BAG3*, *GDF5*, *RHOB*, *RUNX2*, *LGALS3*, and *CDH1* may play a role in regulating ovarian follicle development.

*BAG3*, a member of the BAG family and alternatively known as the Bcl-2-related anti-apoptotic gene 3, encodes for the myosin Z-band protein. Its function as an anti-apoptotic protein is intertwined with various cellular processes, including proliferation, differentiation, apoptosis, migration, and phagocytosis [16,17]. The down-regulation of *BAG3* results in reduced neovascularization [18,19], which could significantly impact the blood supply and nutritional status of ovarian follicles. Studies have indicated that the expression levels of *BAG3* in ovarian cancer tissues may influence the proliferation and apoptosis of cancer cells [20]. In this study, the transcriptional expression of *BAG3* was found to be significantly higher on day 15 compared to days 13 and 11, likely due to its role in follicular nourishment and blood supply, thus exhibiting elevated expression during follicular maturation. These findings offer a robust foundation for further exploration of the role of *BAG3* in ovarian development.

*RHOB*, a member of the Rho family of small GTPases, contributes to ovarian follicle development through a variety of mechanisms, such as regulating cell migration, adhesion, proliferation, differentiation, and apoptosis, as well as modulating dynamic changes in the cytoskeleton [21]. Identified as an innovative genetic marker for angiogenesis and vascular development in porcine follicular granulosa cells [22], *RHOB* demonstrated increased expression on day 15 as the follicles reached maturity, a finding that corroborates previous research on pigs.

*GDF5*, part of the TGF-β superfamily, is crucial for the development of tissues like cartilage and fat. Mutations in *GDF5* are associated with conditions such as apical dysplasia, chondrodysplasia, synovial syndromes, syndactyly, and susceptibility to osteoarthritis [23,24,25,26]. Bahire’s study showed higher *GDF5* expression in ewes with the FecB gene, which is linked to multiple births [27]. Our study found that *GDF5* expression increased significantly by day 15 compared to day 11, hinting at its role in late estrous or follicle maturation. However, more research is needed to fully understand how GDF5 affects ovarian follicle development.

*RUNX2*, a member of the Runt domain family of transcription factors, binds to promoters like StAR and CYP11A1 to regulate hCG-induced progesterone production in granulosa cells [28]. It transcriptionally controls LH-induced Hapln1 expression, enhances granulosa cell viability, reduces apoptosis, and is highly expressed in ovulatory follicle granulosa cells and the oocyte complex [29,30]. Studies indicate that *RUNX2*, a granulosa cell gene regulated by ERβ, plays a significant role in follicle maturation [31]. Transcriptome analysis and fluorescence quantitative validation analysis revealed peak *RUNX2* gene expression on day 13, suggesting that its high expression may improve cell vitality and reduce apoptosis, potentially serving as a marker for follicle growth and providing a theoretical foundation for understanding ovarian follicle development.

The *LGALS3* gene, which encodes galectin-3, contributes to cellular processes such as endothelial cell proliferation and migration, as referenced in [32], and is potentially involved in follicular growth and angiogenesis. Fluctuations in its expression can influence the viability of granulosa cells and apoptotic processes, as indicated in [33]. Galectin-3 is vital for placental function in mice, impacting maternal progesterone levels, as reported in [34]. In pigs, treatment with allylprogesterone (ALT) was shown to up-regulate *LGALS3* in both the ovaries and the uterus, as documented in [35], thereby promoting follicle development and enhancing reproductive performance. In this study, the expression of *LGALS3* on days 13 and 15 was notably higher than on day 11, further confirming this gene’s pivotal role in ovarian growth and follicle maturation.

*CDH1*, a calmodulin family member, is key to cell adhesion, migration, and proliferation [36]. Its loss can cause cell deactivation and migration problems, and it is associated with various cancers [37]. In females, the APC (*CDH1*) complex can regulate cyclin B1 levels, affecting oocyte G2 arrest [38,39], as confirmed by Reis for the first meiotic division in mouse oocytes [40]. Our transcriptional analysis also found no significant difference in *CDH1* expression on days 15 and 11, suggesting that this gene may negatively affect follicle maturation.

## 5. Conclusions

This study identified key genes like *BAG3*, *GDF5*, *RHOB*, *RUNX2*, *LGALS3*, and *CDH1* and significant pathways such as endoplasmic reticulum protein processing, NOTCH signaling, and MAPK signaling as involved in ovarian follicle development in Duolang sheep. RT-qPCR confirmed the differential expression of *BAG3*, *RHOB*, and *RUNX2*. With 422 differentially expressed genes found across three developmental stages, this research provides insights into the molecular mechanisms of follicle development and a basis for enhancing reproductive performance in sheep. These findings also lay the foundation for future gene screening and functional validation of critical reproductive traits.

## Figures and Tables

**Table 1:** Results of pre-processing sequencing data.

Sample ID	Raw Reads	Clean Reads	Clean Bases	Clean Ratio (%)	Q20 (%)	Q30 (%)
DTA1	35,201,732	34,599,992	10.38G	98.29	96.72	95
DTA2	43,245,660	42,453,517	12.74G	98.17	96.76	94.96
DTA3	39,512,871	38,877,267	11.66G	98.39	96.85	95.08
DTB1	42,120,748	41,341,445	12.4G	98.15	96.62	94.86
DTB2	41,952,090	41,237,817	12.37G	98.30	96.1	94.08
DTB3	37,962,740	37,200,417	11.16G	97.99	96.12	94.15
DTC1	38,470,424	37,869,649	11.36G	98.44	96.42	94.56
DTC2	45,956,002	45,135,112	13.54G	98.21	96.8	95.02
DTC3	51,147,006	50,215,954	15.06G	98.18	96.03	93.83

Note: Q20: the percentage of bases with a Phred value greater than 20 in the total bases. Q30: the percentage of bases with a Phred value greater than 30 in the total bases.

**Table 2:** Results of sequencing sequences compared to the reference genome.

Sample ID	Total Reads	Total Map	Unique Map
DTA1	69,199,984	58,278,035 (84.22%)	54,937,151 (79.39%)
DTA2	84,907,034	70,679,122 (83.24%)	66,714,211 (78.57%)
DTA3	77,754,534	65,144,708 (83.78%)	61,556,713 (79.17%)
DTB1	82,682,890	67,937,003 (82.17%)	63,987,224 (77.39%)
DTB2	82,475,634	67,294,692 (81.59%)	63,555,854 (77.06%)
DTB3	74,400,834	61,123,995 (82.15%)	57,632,036 (77.46%)
DTC1	75,739,298	62,363,535 (82.34%)	58,649,112 (77.44%)
DTC2	90,270,224	74,067,730 (82.05%)	69,736,018 (77.25%)
DTC3	100,431,908	79,698,447 (79.36%)	75,107,870 (74.78%)

**Table 3:** Differentially expressed genes associated with follicular development in the ovaries of the Duolang sheep.

Gene Name	Description	DTA/DTB		DTB/DTC		DTA/DTC	
		log2FC	*p*-Value	log2FC	*p*-Value	log2FC	*p*-Value
*BAG3*	BCL2 Associated Athanogene 3	−0.24	<0.05	−1.00	<0.01	−1.25	<0.01
*RHOB*	Ras Homolog Family Member B	−0.37	<0.05	−0.70	<0.01	−1.07	<0.01
*GDF5*	Growth Differentiation Factor 5	0.52	>0.05	−0.84	>0.05	−1.36	<0.01
*LGALS3*	Galectin-3	−0.16	>0.05	−1.31	<0.01	−1.47	<0.01
*RUNX2*	Runt-Related Transcription Factor 2	−1.23	>0.05	1.65	<0.01	0.43	>0.05
*CDH1*	Cadherin 1	1.64	<0.01	−1.31	<0.01	0.34	>0.05

**Table 4:** Primer information on genes.

Gene Name	Sequence (5′-to-3′)	Accession Number	Product Size (bp)
*RUNX2*	F:TGAGCTCCGAAATGCCTCTG	XM_042237136	267
	R:GGATGAGGAATGCGCCCTAA		
*BAG3*	F:AAGCCCAGAAGACGCACTAC	XM_004020234	250
	R:GGTTCTCGATGGGTCATGGG		
*RHOB*	F:GCACGTATGCGCACTCTTTT	NM_001127673	194
	R:AGTACCACTGGATGGGGGAA		
*GAPDH*	F:GCCGCATCCCTGAGACAAG	NM_001190390	113
	R:TGATGGCAACGATGTCCACTT		

## Data Availability

The data set provided in this paper is not easy to obtain because the data is part of the ongoing research and therefore cannot be shared at this time.
